# The influence of high-fat, high-sugar diet and bariatric surgery on HSP70 and HSP90 plasma and liver concentrations in diet-induced obese rats

**DOI:** 10.1007/s12192-019-00976-2

**Published:** 2019-03-06

**Authors:** Dominika Stygar, Bronisława Skrzep-Poloczek, Ewa Romuk, Elżbieta Chełmecka, Jakub Poloczek, Tomasz Sawczyn, Justyna Maciarz, Michał Kukla, Konrad W. Karcz, Jerzy Jochem

**Affiliations:** 1 Department of Physiology, School of Medicine with the Division of Dentistry in Zabrze, Medical University of Silesia, Katowice, Poland; 2 Department of Biochemistry, School of Medicine with the Division of Dentistry in Zabrze, Medical University of Silesia, Katowice, Poland; 3 Department of Statistics, Department of Instrumental Analysis, School of Pharmacy with the Division of Laboratory Medicine in Sosnowiec, Medical University of Silesia, Katowice, Poland; 4 Department of Rehabilitation, 3rd Specialist Hospital in Rybnik, Rybnik, Poland; 5 Department of Gastroenterology and Hepatology, School of Medicine in Katowice, Medical University of Silesia, Katowice, Poland; 6 Clinic of General, Visceral, Transplantation and Vascular Surgery, Hospital of the Ludwig Maximilian University, Munich, Germany

**Keywords:** Bariatric surgery, High-fat, high-carbohydrate diet, HSP70, HSP90, Chaperones, Oxidative stress

## Abstract

Metabolic surgery ameliorates insulin resistance and is associated with long-term, effective weight loss, but the mechanisms involved remain unknown. Here, the duodenal-jejunal omega switch (DJOS) surgery in combination with high-fat, high-carbohydrate diet was performed on diet obese rats and joint effects of bariatric surgery and different dietary patterns on heat shock protein 70 (HSP70) and HSP90 plasma and liver concentrations were measured. We found that plasma and liver levels of HSP70 were lower after DJOS surgery in comparison to the control in the groups of animals kept on control diet (CD) and high-fat, high-sugar diet (HFS) but the postoperative change of the diet led to the increase in HSP70 in plasma and liver concentration in DJOS-operated animals. A high-calorie meal, rich in carbohydrates and fats, significantly increased circulating levels of HSP90, reducing the normalising effect of DJOS. The HFS diet applied during all stages of the experiment led to the higher levels of liver HSP90 concentration. The combination of CD and DJOS surgery was the most efficient in the lowering of the HSP90 liver concentration. The normalisation of circulating levels and liver concentrations of HSP70 and HSP90 may be achieved in a combination of DJOS procedure with a proper dietary plan.

## Introduction

The complex pathophysiology of obesity represents a challenge for the mechanistic understanding of the action of different stimuli on the human body and of their possible role in metabolic distress. Oxidative stress, under the conditions of obesity, is one of the early events in the development of metabolic syndrome (Aroor and DeMarco 2014, Furukawa et al. 2004). Heat shock proteins (HSPs) are a family of stress-responsive proteins that modulate cell function and contribute to protein homeostasis (Atalay et al. 2009, Asea 2008). The HSP70 is a class of highly abundant and ubiquitously expressed chaperones that participate in protein trafficking, early stages of nascent polypeptide folding and the refolding or degradation of aggregated peptide products (Lackie et al. 2017, Bukau et al. 2006). In the situation, when correct folding of the client proteins is not possible, HSP70 association with additional co-chaperones promotes degradation of the misfolded protein (Lackie et al. 2017). Nakhjavani et al. reported previously that the serum level of plasma extracellular HSP70 (eHSP70) was significantly higher in long-term type 2 diabetes mellitus patients in comparison to the newly diagnosed subjects and the higher eHSP70 levels were inversely correlated with fasting blood glucose concentration (Nakhjavani et al. 2010). High HSP70 plasma levels are associated with the inhibition of nitric oxide synthase (NOS) under inflammatory conditions in T2DM (Nakhjavani et al. 2012). HSP70 plasma levels were also increased in diabetic nephropathy, as a response to the oxidative damage (Morteza et al. 2013). Plasma HSP70 concentrations in gestational diabetes correlate well with the levels of glycated haemoglobin (HbA1c) (Garamvölgyi et al. 2015).

In eukaryotic cells, HSP90 clients are, among others, the key mediators of signal transduction and transcriptional regulation (e.g., NF-κB, STATs, p53) and kinases (e.g., Raf/MEK/ERK, PI3K/AKT, p38/MAPK). Thus, HSP90 plays a central role in processes like inflammation, growth, survival, differentiation and apoptosis (Trepel et al. 2010; Pratt and Toft 2003). Increased levels of HSP90 have been observed in malignant cells and inflamed tissues, and that is why HSP90 is particularly studied in the context of the treatment of cancer and autoimmune/inflammatory diseases (Ambade et al. 2014; Shukla and Pitha 2012; Li et al. 2013; Tukaj et al. 2013). HSP90 has been targeted as a new therapeutic approach in hepatocellular carcinoma (HCC) (Breinig et al. 2009) and fibrosis (Myung et al. 2009).

Aside from sustained weight loss, bariatric surgery helps to regulate metabolic processes leading to normalisation of glucose tolerance, insulin sensitisation and increasing energy expenditure (Rubino et al. 2006; Buchwald et al. 2004; Rubino et al. 2010; Stefater et al. 2012). Spectacular effects of bariatric surgery in long-term body weight reduction and glucose tolerance amelioration in obese individuals have stimulated the development of new surgical techniques (Kahn et al. 2006, Karcz et al. 2013). Duodenal-jejunal omega switch (DJOS), as used here, is a bariatric procedure, where a proximal loop duodeno-enterostomy is used to bypass the foregut and allows for direct hindgut stimulation (Nauck 2009; Rubino and Gagner 2002). DJOS as a relatively new technique still needs to be studied in order to assess its long-term pathophysiological effects (Grueneberger et al. 2014; Traverso and Longmire Jr 1978). Therefore, here, an animal model is used in order to address questions regarding the effects of DJOS that could not be pursued experimentally in humans.

Dietary patterns play a significant role in the aetiology and prevention of risk factors like metabolic syndrome, T2DM and obesity (O’Keefe et al. 2008). A high-fat diet leads to disturbances in the expression of inflammatory factors and can deregulate adaptive immunity in obesity (Makki et al. 2013; Rocha et al. 2008). Long-term ingestion with a high-caloric diet interferes with insulin signalling pathways, leads to the increase in peripheral insulin resistance and immune disorders (Cancello and Clément 2006). The high-fat, high-sugar diet (HFS) adopted in this experiment is known to develop hypertension, impaired glucose tolerance, higher abdominal fat deposition, increased abdominal circumference and an altered lipid profile (Poudyal et al. 2010). The digestion of different types of dietary products has different effects on the metabolism, including the expression of *Hsps*. No reports were found in the literature regarding the effects of an HFS diet and bariatric surgery on HSP70 and HPS90 plasma and liver levels. Thus, the aim of this study was to assess the effect of the DJOS procedure combined with a high-fat, high-carbohydrate diet on HSP70 and HSP90 plasma and liver concentrations in diet-induced obese rats.

## Materials and methods

### Animals and diets

All experimental procedures were approved by the Local Ethics Committee, Katowice, Poland (58/2014). The methodology of DJOS was previously described by Karcz et al. 2013 and Stygar et al. 2018. Forty-eight male Sprague–Dawley rats, initially weighing between 250 and 275 g, were purchased from the Center for Experimental Medicine, Medical University of Silesia, Katowice, Poland. Animals were housed in plastic cages under controlled conditions (ambient temperature, 23 °C; 12 h light/dark cycle, lights on 6:00 AM to 6:00 PM). All rats had free access to water and food. The control group was maintained on a professional, regular diet (Provimi Kliba AG, Kaiseraugst, Switzerland, 24% protein, 4.9% fat, 7% crude ashes, 4.7% crude fibre). Obesity was induced by keeping the animals on an HFS diet (HFS 22.0 MJ/kg, 45% fat, 35% carbohydrate and 20% protein (ssniff® EF R/M acc. D12451 (II) mod., Ssniff Spezialdiäten GmbH, Germany) for the period of 2 months before and after the surgery. All rats fasted overnight before surgery.

### Experimental design

**Fig. 1:**
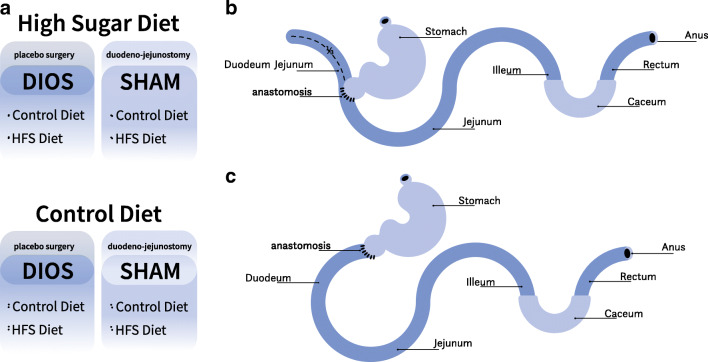
Scheme of the experiment. **a** Dietary patterns used pre- and post-surgery. **b** DJOS procedure. **c** SHAM procedure

After 1 week of acclimatisation, the rats were allocated to the experimental groups: HFS diet (*n* = 28) and control diet (CD) (*n* = 28). The total length of the experiment was 16 weeks. For 8 weeks before and after the DJOS/SHAM surgery, the animals were maintained on their allocated diets. The initial part of the procedure, prior to the surgery, included 8 weeks of maintenance on experimental diets. Following this period, both groups were further split into subgroups, which then underwent two separate types of surgery: SHAM (*n* = 14) or DJOS (*n* = 14, Fig. 1a–c).

For the second stage of the experiment, through the 8 weeks following the surgery, seven animals from the DJOS and SHAM groups were maintained on the same diet as prior to the surgery taking place (HFS/DJOS/HFS, HFS/SHAM/HFS, CD/DJOS/CD, CD/SHAM/CD), and a further seven from each group had an altered diet (HFS/DJOS/CD, HFS/SHAM/CD, CD/DJOS/HFS, CD/SHAM/HFS; Fig. 1a). The number of rats included in the research was minimalised in consideration of the ‘3Rs’ for the humane treatment of animals (Russell and Burch 1959). The survival rate of animals during the experiment and post-surgery was 100%.

### Experimental procedures

The DJOS was performed according to Karcz et al. (Karcz et al. 2013), described in the aforementioned study (Stygar et al. 2018). To perform DJOS, the animals were anaesthetised with 2% isoflurane (AbbVie Deutschland GmbH & Co. KG, Ludwigshafen, Germany) and oxygen flow at 2 l/min under spontaneous breathing. Analgesia with xylazine (5 mg/kg, ip; Xylapan, Vetoquinol Biovet, Poland) and antibiotic prophylaxis with gentamicin (40 mg/ml, Krka, Poland) was applied. In order to gain abdominal access, a midline incision of 3–4 cm was performed, and the total length of the small intestine was determined. The stomach was separated from the duodenum at the point just below the pylorus and the position of anastomosis was defined as being at 1/3 of the total small bowel length. The jejunum was anastomosed via end-to-side duodeno-enterostomy in order to restore the physiological conduit of the food passage, excluding the duodenum and parts of the small intestine. The remaining duodenal stump was closed using PDS 6/0 (Ethicon, US, LLC). Mesenteric openings were closed with PDS 6/0 (Ethicon, US, LLC). In the SHAM-operated animals, reanastomosis of the gastrointestinal tract was performed at the corresponding sites where enterotomies were performed for the duodenojejunostomy, thereby maintaining continuity of the food passage through the bowel (Fig. 1b, c). For DJOS and SHAM protocols, postoperative analgesia was performed using carprofen (4 mg/kg, sc; Rimadyl, Pfizer, Switzerland) for three consecutive days after the surgery.

### Tissue collection and HSP70 and HSP90 ELISA analysis

At the end of the 8th week after surgery, corresponding to the 16th week of the experiment, the tissue samples for HSP measurements were collected. The animals were anaesthetised with 2% isoflurane and oxygen flow at 2 l/min under spontaneous breathing. Analgesia was conducted with xylazine. Blood samples were collected from the abdominal aorta, using tubes containing 10 μl EDTA (Sigma-Aldrich, St. Louis, MO). After centrifugation at 4000 rpm for 10 min at 4 °C, plasma samples were collected, snap-frozen in liquid nitrogen and stored at − 80 °C until analyses were performed.

Liver tissues were harvested and the animals were subjected to euthanasia. Tissue samples were prepared by homogenisation and sonification (15 s) on ice, in a tissue cell lysis buffer containing protease inhibitors (Gold Bio Technology, USA, MO). After that, the homogenates were centrifuged at 15,000*g* for 15 min at 4 °C. Homogenates were snap-frozen in liquid nitrogen and stored at − 80 °C until further analysis. HSP70 plasma and liver concentrations were measured using the rat HSP70 enzyme-linked immunosorbent assay (ELISA) Kit (Enzo Life Sciences, Inc., NY; ADI-EKS-715). The smallest concentration of a HSP70 that could be reliably measured by selected analytical Kit was 90 pg/ml, with detection range 0.20–12.5 ng/ml; ADI-EKS-700B, minimum sensitivity 200 pg/ml (detection range 780–50,000 pg/ml) according to the manufacturer’s instructions. HSP90 plasma and liver concentrations were measured using the rat HSP90 ELISA (Wuhan USCN Business Co., Ltd. Product no SEA823Ra). The smallest concentration of a HSP90 that could be reliably measured by selected analytical Kit was 0.055 ng/ml, with detection range 0.156-10 ng/ml. Each experiment was performed in duplicate.

## Statistical analysis

Statistical analysis was performed using STATISTICA 13.1 PL (StatSoft, Cracow, Poland). All tests were two-tailed and statistical significance was set at a *p* value below 0.05. Interval data were expressed as mean value ± standard deviation. Distribution of variables was evaluated by the Shapiro–Wilk test and the quantile–quantile plot; homogeneity of variances was assessed by Levene’s test. For comparison of data, the two-way parametric ANOVA with post hoc contrast analysis was used.

## Results

Table 1 shows the measured plasma concentrations of HSP70 and HSP90 and the HSP70 and HSP90 liver concentrations of rats that underwent DJOS or SHAM surgery. For the analysed HSP70, the plasma concentrations and liver levels were not related to DJOS and SHAM surgery, and were related to the interaction between surgery and the type of diet applied before/after surgery. Table 1 also shows differences in HSP90 plasma and the liver tissues of animals after both types of surgery. The impact of the type of surgery on HSP90 plasma and HSP90 liver levels is different than in the HSP70 analysis where the impact of surgery was not observed. In all analysed DJOS study groups, the HSP90 levels were significantly different, except for the comparison of the groups where diet was changed after surgery (CD/HFS and HFS/CD). In those groups, HSP90 plasma levels were similar and significantly higher when compared to the CD/CD group but significantly lower when compared to the HFS/HFS group. A similar situation is observed in SHAM-operated groups where significant differences between the groups were observed. Significant differences were deduced from two-way ANOVA analysis between the type of surgery, groups and interaction between group and operation type. When the two-way analysis of variance shows that one of the main analysed factors is statistically significant, and when also—but not necessarily—an interaction between two main factors occurs, then contrast analysis can be performed. This means that we can compare each subclass of the first factor between groups defined by the first factor (*p* value for comparisons between types of operation, SHAM and DJOS), and each subclass of the second factor between groups defined by the first factor (*p* value for comparisons between diets, i.e. HF/HF; HF/CD; CD/HF; CD/CD). Multiple comparisons, in contrast analysis of HSP plasma and liver levels in DJOS and SHAM-operated groups in relation to the diet used before and after surgery, are presented in Table 2. Column 1 shows a comparison between DJOS and SHAM surgery associated with different diets, column 2 shows comparisons between dietary groups of DJOS animals, and column 3 shows comparisons between dietary groups of SHAM animals.

**Table 1:** HSP70 and HSP90 plasma levels and liver concentration 8-week postoperatively DJOS (1st column) and SHAM (2nd column) surgery, subjected to different dietary patterns and intergroup comparison between DJOS and SHAM study groups (3rd column) using descriptive statistics and results of two-way analysis of variance

	DJOS (1st column)				SHAM (2nd column)				*p* ANOVA (3rd column)		
	CD/CD	CD/HFS	HFS/CD	HFS/HFS	CD/CD	CD/HFS	HFS/CD	HFS/HFS	Group	Op.	Int.
HSP70 plasma (ng/ml)	0.69 ± 0.06	3.92 ± 0.39	3.74 ± 0.70	1.30 ± 0.41	1.47 ± 0.08	1.87 ± 0.13	2.40 ± 0.54	3.75 ± 0.20	*< 0.001*	0.695	*< 0.001*
HSP70 liver tissue (ng/mg protein)	1.73 ± 0.27	3.56 ± 0.11	4.09 ± 0.44	2.14 ± 0.35	3.20 ± 0.07	2.67 ± 0.45	3.18 ± 0.44	2.48 ± 0.34	*< 0.001*	0.975	*< 0.001*
HSP90 plasma (ng/ml)	0.54 ± 0.11	2.36 ± 0.26	2.31 ± 0.31	3.11 ± 0.99	1.58 ± 0.53	2.60 ± 0.27	4.09 ± 0.93	3.17 ± 0.88	*< 0.001*	*< 0.001*	*< 0.01*
HSP90 liver tissue (ng/mg protein)	0.25 ± 0.07	1.64 ± 0.39	3.30 ± 0.25	3.57 ± 0.58	2.23 ± 0.44	4.09 ± 0.56	3.96 ± 1.05	9.86 ± 0.90	*< 0.001*	*< 0.001*	*< 0.001*
Body weight (g)	361.1 ± 29.0	383.7 ± 17.3	392.7 ± 15.7	435.4 ± 21.8	382.3 ± 47.7	376.4 ± 48.3	427.0 ± 30.5	473.0 ± 40.7	*< 0.001*	*< 0.05*	0.289

Mean ± standard deviation or median (lower–upper quartile); the statistical significance was set at a *p* value below 0.05

Op., operation type; Int., interaction between group and operation type

**Table 2:** Multiple comparisons in contrast analysis. Column 1: intergroup comparisons between HFS/HFS, CD/HFS, HFS/CD and CD/CD groups DJOS versus SHAM. Column 2: intragroup comparisons between HFS/HFS, CD/HFS, HFS/CD and CD/CD groups after DJOS surgery. Column 3: intragroup comparisons between HFS/HFS, CD/HFS, HFS/CD and CD/CD groups after SHAM surgery

Post hoc	DJOS vs SHAM				DJOS						SHAM					
	1: CD/CD	2: CD/HFS	3: HFS/CD	4: HFS/HFS	1 vs 2	1 vs 3	1 vs 4	2 vs 3	2 vs 4	3 vs 4	1 vs 2	1 vs 3	1 vs 4	2 vs 3	2 vs 4	3 vs 4
HSP 70 plasma (ng/ml)	*< 0.01*	*< 0.001*	*< 0.001*	*< 0.001*	*< 0.001*	*< 0.001*	*< 0.01*	0.386	*< 0.001*	*< 0.001*	0.073	*< 0.001*	*< 0.001*	*< 0.05*	*< 0.001*	*< 0.001*
HSP 70 liver tissue (ng/mg protein)	*< 0.001*	*< 0.001*	*< 0.001*	0.097	*< 0.001*	*< 0.001*	*< 0.05*	*< 0.05*	*< 0.001*	*< 0.001*	*< 0.05*	0.929	*< 0.001*	*< 0.05*	0.344	*< 0.01*
HSP 90 plasma (ng/ml)	*< 0.01*	0.518	*< 0.001*	0.858	*< 0.001*	*< 0.001*	*< 0.001*	0.880	*< 0.05*	*< 0.05*	*< 0.01*	*< 0.001*	*< 0.001*	*< 0.001*	0.122	*< 0.05*
HSP 90 liver tissue (ng/mg protein)	*< 0.001*	*< 0.001*	0.069	*< 0.001*	*< 0.001*	*< 0.001*	*< 0.001*	*< 0.001*	*< 0.001*	0.447	*< 0.001*	*< 0.001*	*< 0.001*	0.712	*< 0.001*	*< 0.001*
Body weight (g)	0.246	0.687	0.063	*< 0.05*	0.216	0.086	*< 0.001*	0.619	*< 0.01*	< 0.05	0.746	*< 0.05*	*< 0.001*	< 0.01	*< 0.001*	*< 0.05*

The statistical significance was set at a *p* value below 0.05

### HSP70 plasma and liver concentrations

**Fig. 2:**
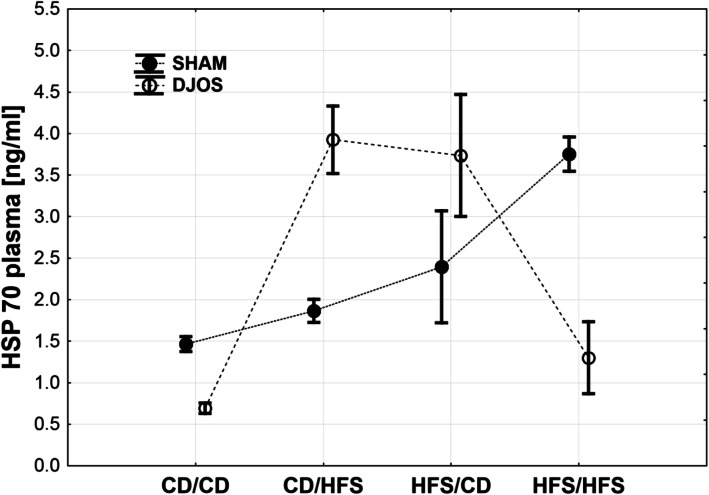
Comparison of HSP70 plasma levels (ng/ml) in SHAM and DJOS-operated groups subjected to the different dietary patterns pre- and post-surgery. Abbreviations: CD, control diet; HFS, high-fat, high-sugar diet. Vertical lines depict 95% confidence interval

The plasma levels and liver concentrations of HSP70 were significantly related to the type of diet and interaction between the diet and surgery but not to the surgery itself (Fig. 2; Tables 1 and 2).

#### DJOS vs SHAM

Significant differences in the HSP70 plasma levels were observed for all analysed study groups (Fig. 2; Tables 1 and 2). For HSP70 liver concentration, significant differences between CD/CD, CD/HFS and HFS/CD groups were observed (Fig. 2; Tables 1 and 2).

#### DJOS surgery

**Fig. 3:**
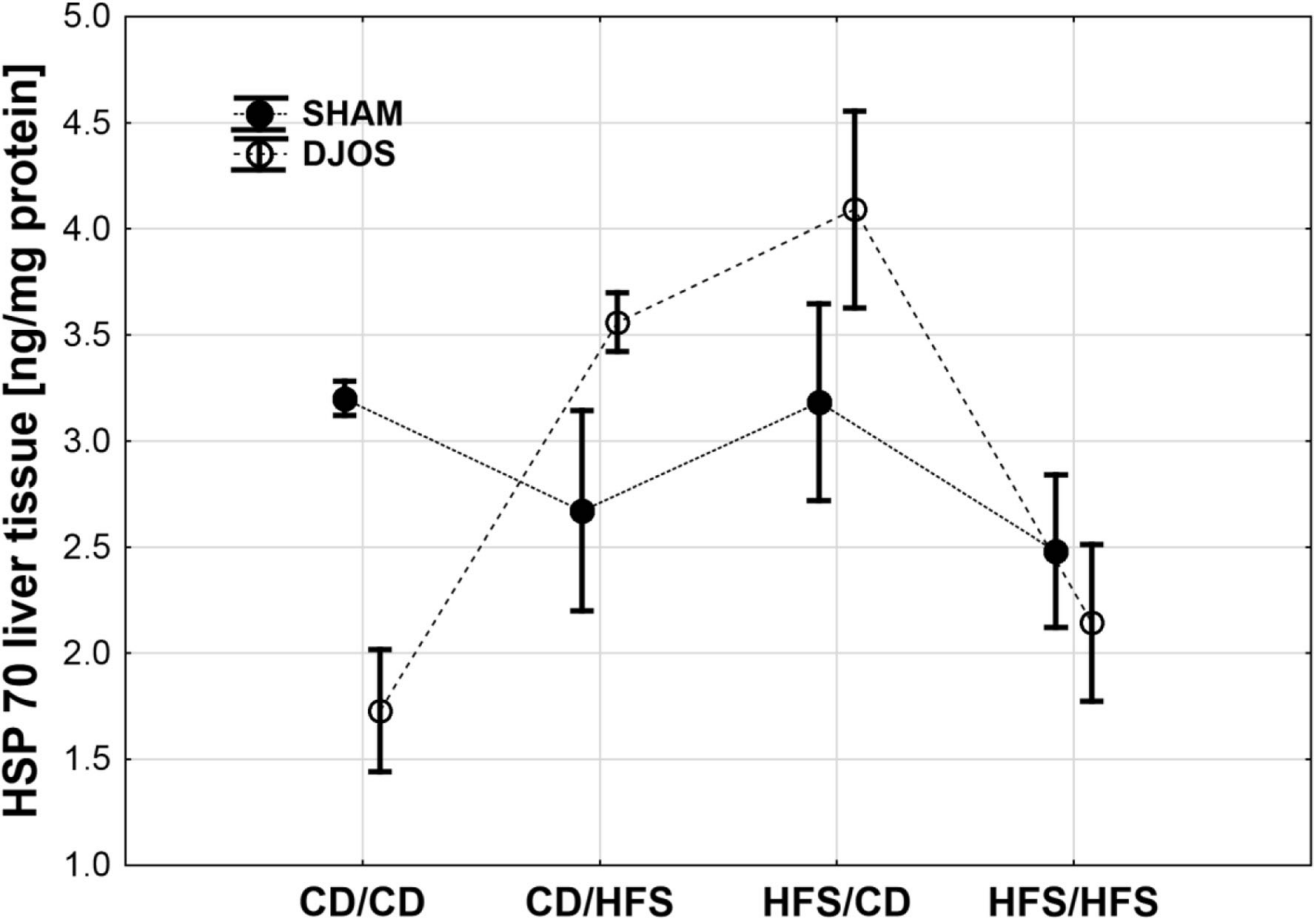
Comparison of HSP70 liver concentration (ng/mg) in SHAM and DJOS-operated groups subjected to the different dietary patterns pre- and post-surgery. Abbreviations: CD, control diet; HFS, high-fat, high-sugar diet. Vertical lines depict 95% confidence interval

After DJOS surgery, the plasma level and liver concentrations of HSP70 were significantly higher in the groups, where the diet was changed after the surgery (CD/HFS and HFS/CD) when compared to the groups, which remained on an unchanged diets (HFS/HFS, CD/CD) (Figs. 2 and 3; Tables 1 and 2).

#### SHAM surgery

In the SHAM-operated animals, the level of HSP70 in plasma was significantly increasing in the following groups: CD/CD vs CD/HFS vs HFS/CD vs HFS/HFS (Fig. 2; Tables 1 and 2), with the lowest values of plasma HSP70 in the CD/CD group (Fig. 2; Tables 1 and 2). The HSP70 liver concentrations were the highest in the CD/CD and HFS/CD groups. The value of this parameter was significantly reduced in the CD/HFS group when compared to CD/CD one. The liver HSP70 concentration was significantly higher in HFS/CD group when compared to CD/HFS and HFS/HFS ones (Fig. 3; Tables 1 and 2).

### HSP90 plasma concentrations

The plasma and liver concentrations of HSP90 were significantly dependent on the type of diet used in the experiment, the type of surgery and interactions between both types of parameters (Table 1).

#### DJOS vs SHAM

**Fig. 4:**
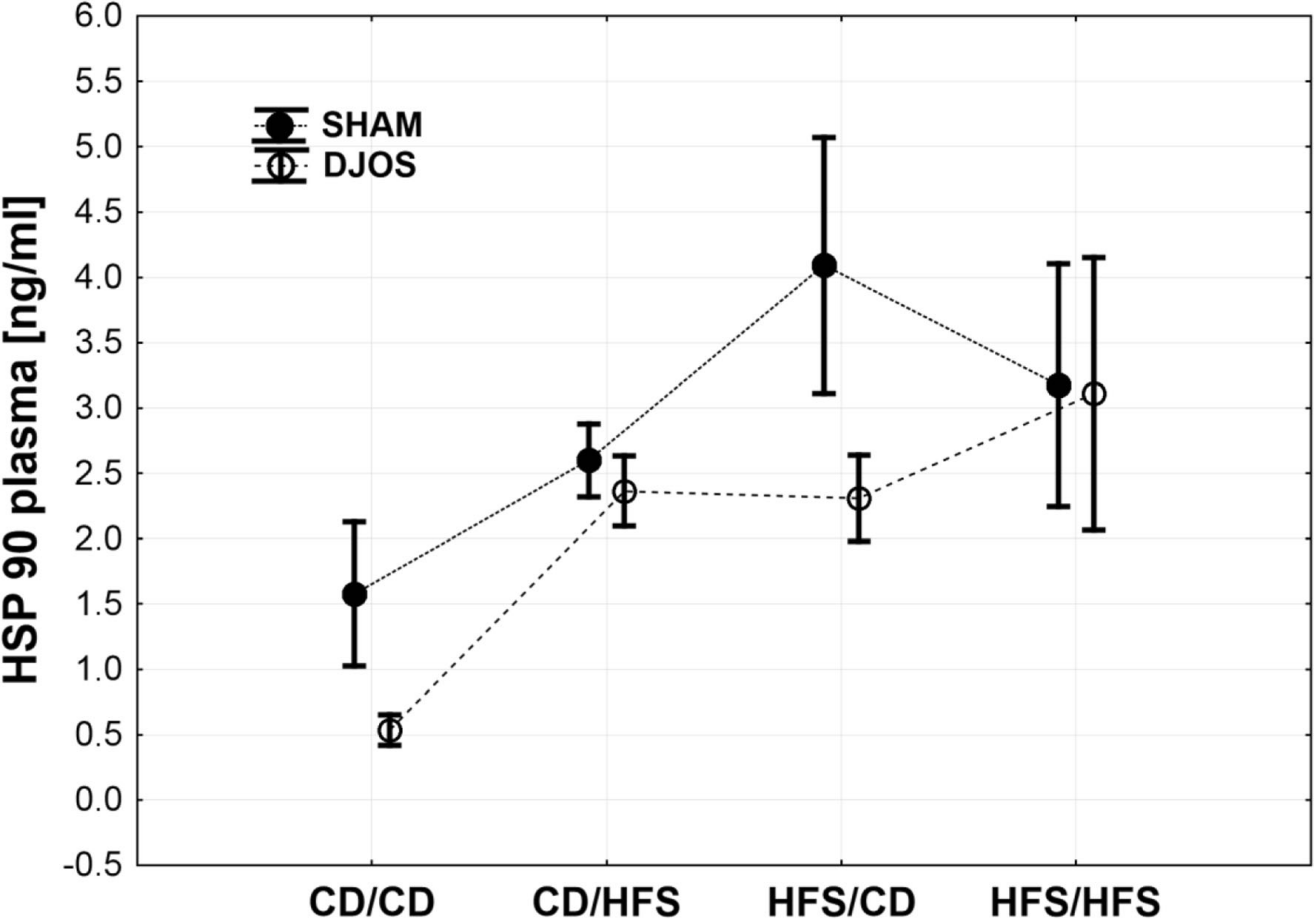
Comparison of HSP90 plasma levels (ng/ml) in SHAM and DJOS-operated groups subjected to the different dietary patterns pre- and post-surgery. Abbreviations: CD, control diet; HFS, high-fat, high-sugar diet. Vertical lines depict 95% confidence interval

A comparison of HSP90 plasma concentrations collected from DJOS and SHAM-operated animals shows significant differences between CD/CD and HFS/CD groups, where values of HSP90 were higher in the samples collected from the SHAM-operated animals (Fig. 4; Tables 1 and 2).

#### DJOS surgery

After DJOS surgery, the lowest values of plasma HSP90 levels were observed in the CD/CD group when compared to other diet and surgery combinations (Fig. 4; Tables 1 and 2). The combinations of HFS diet (HFS/CD, HFS/HFS) applied before and after the surgery led to an increase in HSP90 plasma levels when compared to the CD/CD and CD/HFS groups.

#### SHAM surgery

In the SHAM-operated animals, similarly to the DJOS groups, a change of the diet from CD before or after surgery to the HFS diet led to a significant increase in plasma HSP90 levels (Fig. 4; Tables 1 and 2).

### HSP90 liver concentrations

The HSP90 liver concentration was related to the type of studied diet, type of surgery and interaction between those parameters (Table 1).

#### DJOS vs SHAM

The liver concentration of HSP90 was significantly higher in SHAM-operated animals in comparison to DJOS-operated rats except for HFS/CD groups (Table 1).

#### DJOS surgery

**Fig. 5:**
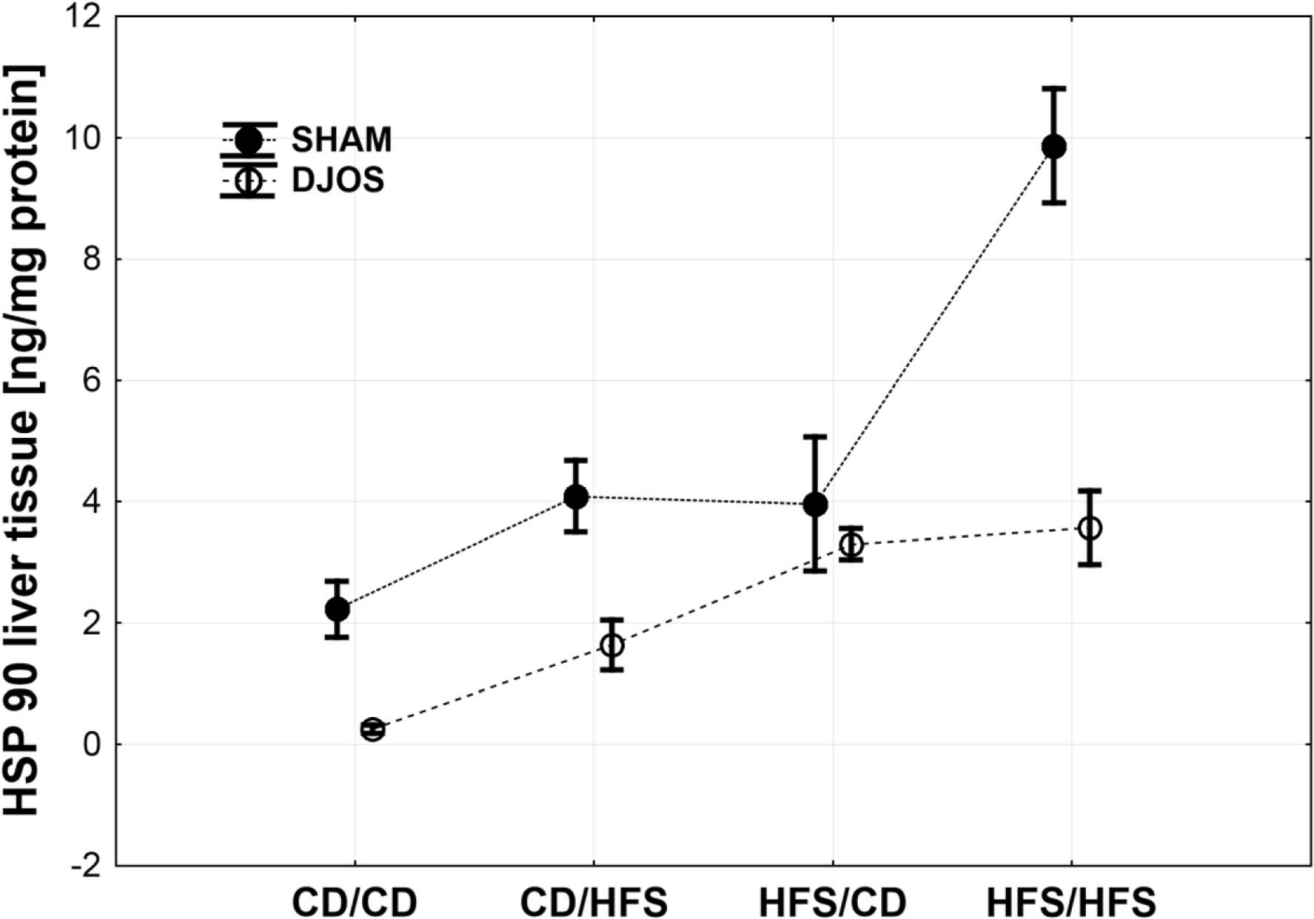
Comparison of HSP90 liver concentration (ng/mg) in SHAM and DJOS-operated groups subjected to the different dietary patterns pre- and post-surgery. Abbreviations: CD, control diet; HFS, high-fat, high-sugar diet. Vertical lines depict 95% confidence interval

In the DJOS-operated animals, the change of the dietary pattern from CD to HFS after the surgery or application of an HFS diet before and after surgery led to a significant increase in the HSP90 liver concentration (Fig. 5; Tables 1 and 2). The lowest HSP90 level was observed in the combinations with CD before/after the surgery, and the highest in the presence of an HFS diet during all experiments (Fig. 5; Tables 1 and 2).

#### SHAM surgery

A similar pattern of HSP90 liver concentrations was also observed in SHAM-operated groups, with the lowest values in the CD/CD group and increasing from the CD/HFS to HFS/CD and to HFS/HFS groups (Fig. 5; Tables 1 and 2). There were no significant differences when comparing the CD/HFS and HFS/CD groups.

### Body weight

The type of dietary pattern and type of surgery applied had a significant impact on the body weight of animals from the analysed study groups (Tables 1 and 2).

#### DJOS vs SHAM

**Fig. 6:**
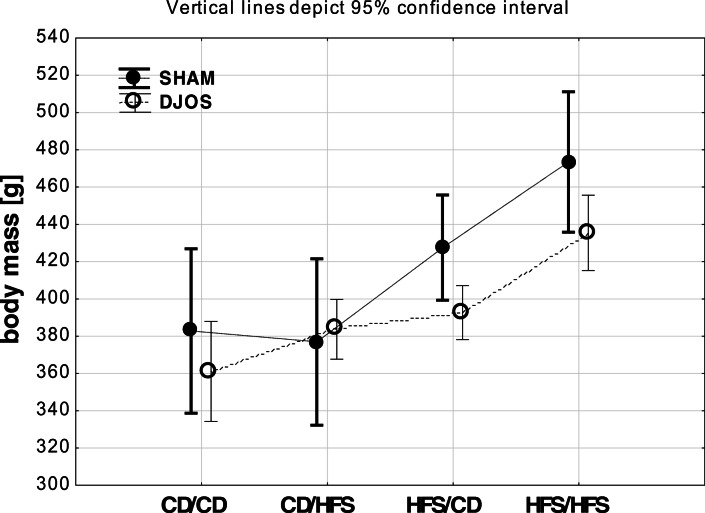
Body mass (g)

All the groups that consumed an HFS (HFS/HFS) diet had a significant increase in body weight when compared to the control group (CD/CD). The body weight of animals maintained on an HFS diet before and after the surgery was significantly higher in the SHAM-operated animals when compared to DJOS (Fig. 6; Tables 1 and 2).

#### DJOS surgery

After DJOS, the body weight in HFS/HFS group was significantly higher in relation to CD/CD animals. The dietary pattern which included a CD before and an HFS diet after the surgery influenced the body weight of DJOS-operated animals in comparison to the HFS/HFS diet profile.

#### SHAM surgery

After SHAM surgery, the animals kept on HFS/HFS expressed the highest body weight when compared with the CD/CD, CD/HFS and HFS/CD groups. The usage of HFS before the surgery significantly increased the body mass in comparison to the CD/CD group in SHAM-operated animals.

As we reported before (Stygar et al. 2018), a change of diet to a more caloric one after DJOS surgery did not lead to significant weight loss when the surgery was not combined with gastric restriction. In our study, we observed that a high-caloric diet reduced the effects of the surgery, in contrast with other reported types of surgical procedures (Mosinski et al. 2016).

## Discussion

The objective of this study was to assess the effect of bariatric surgery combined with different dietary patterns on HSP70 and HSP90 plasma and liver concentrations. A combination of high-fat, high-sugar diet was used to mimic human nutritional patterns that have been associated with the development of the metabolic syndrome, obesity and T2DM. We report that (i) plasma and liver HSP70 levels were not related to the type of surgery but were related to the type of diet and interaction between diet and surgery; (ii) HSP70 concentrations were lower after DJOS bariatric surgery in comparison to the SHAM surgery in the groups of animals kept on CD and HFS diet pre- and postoperatively; (iii) the change of the diet, regardless of the type of diet (CD/HFS or HFS/CD), was a strong stimulus for HSP70 induction in plasma and liver tissue in DJOS-operated animals when compared to SHAM groups; (iv) plasma and liver HSP90 levels are related to the type of diet and type of surgical intervention; (v) the DJOS procedure reduced HSP90 plasma levels when combined with the CD before and/or after the surgery, and in the presence of the HFS/CD dietary pattern; (vi) a high-calorie meal, rich in carbohydrates and fats significantly increased plasma levels of HSP90 in the CD/HFS, HFS/CD and HFS/HFS groups, reducing the normalising effect of DJOS; (vii) the DJOS surgery and CD diet showed a strong reductive impact on HSP90 liver concentration when compared to the SHAM-operated animals; (viii) the HFS diet applied during all stages of the experiment led to the highest levels of liver HSP90 concentration; (ix) the interaction between CD and DJOS surgery was the most efficient at lowering the HSP90 liver concentration in comparison with other dietary patterns.

The present study compares the joint therapeutic responses from the combination of diets and bariatric surgery measured by HSP70 and HSP90 levels. The obtained results suggest that the combination of DJOS and a change of the diet was a strong stimulus for HSP70 level induction in plasma and liver tissue. In our previous study, we reported increased HSP70 plasma levels after the ileal transposition (IT) in comparison to SHAM-operated, diabetic fatty Zucker rats (Stygar et al. 2015). Angelini and co-workers showed that duodenal-jejunal bypass surgery leads to a decrease in levels of plasma HSP70 in obese, insulin-resistant (IR) and nonalcoholic fatty liver disease (NAFLD) subjects (Angelini et al. 2018). Serum HSP70 and plasma eHSP70 levels are known to be elevated in diabetic patients in comparison to normal subjects and correlate with diabetes duration (Nakhjavani et al. 2010). High HSP70 plasma levels are also associated with inflammatory and oxidative stress conditions in T2DM (Nakhjavani et al. 2012, Morteza et al. 2013). Circulating HSP70 levels in gestational diabetes correlate with the levels of glycated haemoglobin (HbA1c) (Garamvölgyi et al. 2015). Alemi and co-authors (Alemi et al. 2018) showed a positive relation between eHSP70 and HOMA-IR but not BMI in patients with T2DM, regardless hs-CRP levels and obesity. These results suggest the role of eHSP70 in the pathogenesis of T2DM and insulin resistance. Angelini et al. 2018 presented a study where a high-energy, high-carbohydrate and high-fat diet can trigger hypersecretion of heat shock proteins from the small intestine that can lead to insulin resistance through inhibition of Akt phosphorylation, insulin-mediated GLUT translocation on the cell membrane and ectopic intracellular fat accumulation with NAFLD development. Heydari et al. 1993 reported that the induction of hsp70 synthesis, mRNA levels and nuclear transcription were significantly higher in hepatocytes isolated from old rats fed the calorie-restricted diet than in hepatocytes isolated from old rats fed ad libitum. Since those two studies of Angelini et al. (2018) and Heydari et al. (1993) were conducted in vivo and in vitro, different parameters were measured. We would suggest that both types of described situations: the caloric limitation and a high-energy diet are strong stressors which lead to the recognised answer in the antioxidant defence system, measured by HSPs levels or *hsps* expression. This can be understood as a type of adaptive reaction, which leads to compensation.

Here, we report that the plasma and liver HSP70 levels were not related to the type of surgery SHAM or DJOS; nevertheless, they were related to the type of diet and the interaction between surgery and dietary pattern. It was observed that the animals, which remain on the same type of diet before and after DJOS surgery (CD/CD, HFS/HFS), showed lower plasma and liver HSP70 concentrations in comparison to groups where the diets were changed after DJOS surgery (CD/HFS and HFS/CD). We suggest that the change of diet, and hence the consumption of a different types of nutrients, influenced the biochemical pathways, causing perturbations and irregularities in substrate catabolism, which is also manifested in HSP production. Nevertheless, in order to confirm this thesis, additional assessments should be provided.

The limitation in caloric supply has been consistently recognised to prolong the life of mammals. Based on the fact that caloric restriction increases the longevity of rodents by altering the ageing process and augments the maximum survival of animals, Heydari et al. reported that reduction of the caloric intake increased *hsp70* gene expression in isolated hepatocytes of old rats, reversing the age-related deficit in the ability of the hepatocytes to respond to heat stress (Heydari et al. 1993). de Moura and co-authors presented a study, where the consumption of whey protein in the hydrolysed form (WPH), used as a source of protein, was able to increase the expression of *hsp70* in the lungs, soleus and gastrocnemius muscles of rats subjected to thermal stress from exercise (Moura et al. 2013). The HSPs family is highly expressed under stress conditions that augment cell tolerance and reduces the adverse effects of sudden, strong and negative stimulation. An antioxidative/prooxidative imbalance caused by a high-fat diet or changes in the nutritional status of diet deficiency, as well as a plethora of selected nutrients, influence the HSP70 and HSP90 concentrations. Changes in the Se, Mn and glutamine levels increased the quantity of *hsp70* and *hsp90* expression. Oral supplementation with arginine enhanced muscle *hsp70* and *hsp90* expression in rats when compared to leucine, isoleucine or valine, under stress conditions produced by exercise (Moura et al. 2018). In our study, the lowest plasma levels of HSP70 and HSP90 were in the groups submitted to unchanged CD or HFS diet during all stages of the experiment. This goes along with results showing that low levels of HSP expression occur in normal physiological conditions (constitutive expression), making up 5–10% of the total protein content in healthy growth conditions (Whitley et al. 1999). It is also known that diet-induced obesity can reduce the expression of HSPs, while the increase of HSP expression can prevent or reduce the damage caused by obesity, promoting an improvement in insulin sensitivity, glucose tolerance, inflammation reduction and an increase in insulin signalling (Henstridge et al. 2014, Chung et al. 2008, Gupte et al. 2009; Moura et al. 2018). These scientific reports regarding HSP levels and hsps gene expression in different pathological situations and stimulation are many times confusing and contradictory. We assume that the answer of the system measured by HSPs depends on the intensity of the stimulation, its duration and character of the general stressor. Thus, different and even contradictory results may be obtained and in order to achieve proper understanding and interpretation of this phenomenon other, more detailed study needs to be performed. Our study suggests that animals exposed to the change of diet after DJOS, but not SHAM, expressed the highest capacity for induction of HSP70, which is a highly active protein and complementary to the antioxidant system, when compared to animals maintained on the same type of diet before and after the surgery (Silver and Noble 2012). In relation to the control groups, the HSP70 plasma levels were significantly increased when the HFS diet was used before and/or after surgery. It was indicated that HSP70 is strongly induced by different types of stress, while its expression is very low or undetectable under conditions of normal homeostasis (Rohde et al. 2005). We would predict that the change of the diet per se and thereby the intake of the different types of nutrients together with DJOS procedure affects the biochemical pathways, leading to increased stress and changes in substrate catabolism, which is also associated with the induction of HSP70. We assume that the exclusion of the proximal part of the small intestine, performed in DJOS, may be more influential than a SHAM procedure. It can be suggested that the DJOS procedure stimulated the blood and liver cells’ protective mechanisms, measured by HSP70 levels, in strong relation to the type of nutritional pattern applied before and after bariatric surgery. Modification of the diet from CD to HFS and vice versa may be considered a less suitable method of treatment and remaining on the stable diet, preferably the control one following bariatric surgery would be suggested. We also showed that exposure to the type of the surgical stress applied in this study did not influence HSP70 plasma and liver concentrations, and interaction between the type of surgery and diet was strong drivers regulating HSP70 response.

HSP90 is a chaperone protein, constitutively expressed in eukaryotic cells, and crucial for its physiological activity. It is estimated that HSP90 represents 1 to 2% of cell proteins in physiological conditions and increases significantly in cells exposed to heat and a variety of other stressful stimuli (Garrido et al. 2001, 47 Csermely et al. 1998). HSP90 is a unique and central component of machinery that comprises a large number of cofactors, which bind reversibly to HSP90, thus establishing a flexible unit for the conformational control of many different cellular proteins (Schopf et al. 2017). This control includes protein function and activity by facilitating protein folding, the binding of ligands to their receptors or targets, or the assembly of multiprotein complexes (Schopf et al. 2017). Several studies showed that HSP90 can be overexpressed in pathological conditions such as plaques of atherosclerosis and alcoholic liver disease (Ambade et al. 2014). HSP90 is found to be elevated in patients with NAFLD (Rodríguez-Suárez et al. 2010). HSP90 inhibition reduces oxidative stress responses (Madrigal-Matute et al. 2012), indicating that HSP90 inhibitors can be used as therapeutic agents for potential treatment. In the present study, we show that the levels of HSP90 in plasma and the liver tissue of rats were related to the type of diet, surgery protocol and interaction between both parameters. The HSP90 plasma and liver levels were higher in the control, SHAM-operated animals when compared to the DJOS procedure. Nevertheless, significantly higher plasma HSP90 levels were observed in the SHAM CD/CD and HFS/CD groups. The baseline of HSP90 levels was significantly increased after SHAM surgery in comparison to DJOS. In our previous studies (Skrzep-Poloczek et al. 2018), we reported that the antioxidant enzymatic systems represented by glutathione peroxidase (GPX), catalase (CAT), superoxide dismutase (CuZnSOD) and nonenzymatic malondialdehyde (MDA) showed a significantly lower level of activity and concentration in the soleus muscle after DJOS surgery in comparison to SHAM-operated animals, and the glutathione reductase (GR) showed significantly increased activity after DJOS in relation to SHAM operation. This may suggest a strong impact of DJOS surgery on the dynamics of antioxidative/oxidative processes. In that study, a change in diet, regardless of the type of diet, stimulated oxidative stress (OS) in DJOS-operated rats. The same type of diet before and after surgery, which was HF/HF and CD/CD, induced OS less than a change in dietary pattern from HF to CD or from CD to HF.

In this study, we would also suggest the potential effect of DJOS in declining the induction of HSP90 levels. The data presented here revealed that the change of the dietary pattern combined with the surgery increased HSP90. The highest impact on HSP90 plasma and liver concentrations came from the HFS diet, both after DJOS and SHAM procedures. Nonetheless, the DJOS procedure was less prone to this amelioration in comparison to the SHAM operation.

These results confirm previous observations that a high-energy, high-carbohydrate and high-fat diet leads to a significant increase in the secretion of HSPs, which can trigger insulin resistance via inhibition of Akt phosphorylation and insulin-mediated GLUT translocation on the cell membrane and ectopic intracellular fat accumulation with NAFLD development (Angelini et al. 2018).

## Conclusions

Bariatric procedures are commonly used in the treatment of obesity and T2DM. Nevertheless, the long-lasting effects of these procedures depend on many factors, among which the diet is one of the most powerful drivers. In order to achieve the expected normalisation of plasma and liver concentrations of HSP70 and HSP90, the DJOS surgery should be applied along with a proper dietary plan. The presence of an HFS diet and the change of the once inducted dietary patterns lead to an increase in HSP70 and HSP90 levels.

## Data Availability

The original data is available after contact with the corresponding author.
